# ACSL4 accelerates osteosarcoma progression via modulating TGF-β/Smad2 signaling pathway

**DOI:** 10.1007/s11010-024-04975-5

**Published:** 2024-04-02

**Authors:** Xiaofeng Li, Qianfen Chen, Duo Zhao, Jianshi Tan, Rongbo Liao, Yurong Gu, Jinwei Zhu, Huying Zhang, Jian Xie, Lu Chen

**Affiliations:** 1 Department of Spine and Osteopathy Surgery, The Second Affiliated Hospital of Guangxi Medical University, Nanning, 530007 Guangxi China; 2 Department of Orthopaedics, The Second Affiliated Hospital of Nanchang University, No.1, Minde Road, Nanchang City, 330006 Jiangxi Province China

**Keywords:** Osteosarcoma, ACSL4, Cell proliferation, Cell apoptosis, TGF-β signaling pathway

## Abstract

Osteosarcoma (OS) is a malignant bone sarcoma arising from mesenchymal stem cells. The biological role of Acyl-CoA synthetase long-chain family member 4 (ACSL4), recently identified as an oncogene in numerous tumor types, remains largely unclear in OS. In this study, we investigated the expression of ACSL4 in OS tissues using immunohistochemistry staining (IHC) staining of a human tissue microarray and in OS cells by qPCR assay. Our findings revealed a significant up-regulation of ACSL4 in both OS tissues and cells. To further understand its biological effects, we conducted a series of loss-of-function experiments using ACSL4-depleted MNNG/HOS and U-2OS cell lines, focusing on OS cell proliferation, migration, and apoptosis in vitro. Our results demonstrated that ACSL4 knockdown remarkably suppressed OS cell proliferation, arrested cells in the G2 phase, induced cell apoptosis, and inhibited cell migration. Additionally, a subcutaneous xenograft mice model was established to validate the in vivo impact of ACSL4, revealing ACSL4 silencing impaired tumor growth in the OS xenograft mice. Additionally, we discovered that ACSL4 could regulate the phosphorylation level of Smad2 through cooperative interactions, and treatment with a TGF-β inhibitor weakened the promoting effects of ACSL4 overexpression. In short, ACSL4 regulated OS progression by modulating TGF-β/Smad2 signaling pathway. These findings underscore ACSL4 as a promising therapeutic target for OS patients and contribute novel insights into the pathogenesis of OS.

**Graphical abstract:**

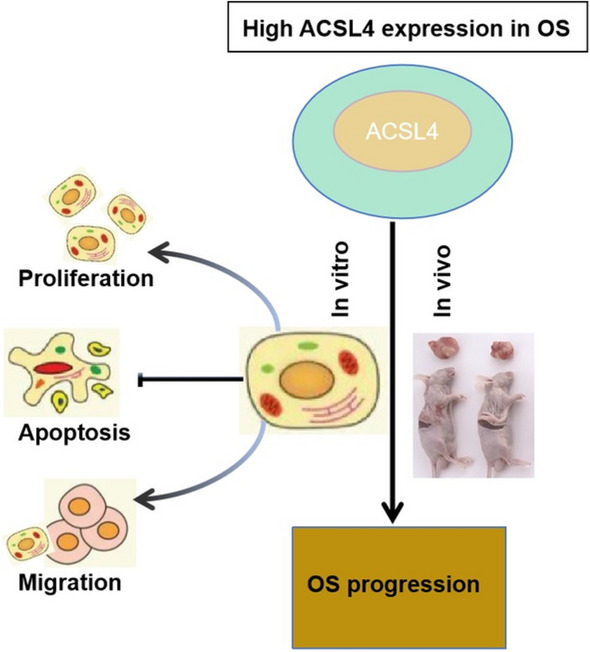

**Supplementary Information:**

The online version contains supplementary material available at 10.1007/s11010-024-04975-5.

## Introduction

Osteosarcoma (OS) is a primary malignant bone tumor deriving form mesenchymal stem cells [1]. It is characterized by high invasive and metastatic potential and has a high incidence in children and adolescence [2]. Clinically, the treatment of OS mainly relies on surgery followed by radiation therapy or adjuvant chemotherapy [3]. Unfortunately, the effective treatment of OS remains a significant challenge due to tumor recurrence, drug resistance, and enhanced invasion and metastasis [4]. Therefore, there is urgent to develop new treatment strategies for OS. Recent advancements in research have shown that molecular-targeted therapies have changed the landscape of cancer treatments, and identification of genetic markers have offered promising prospects for better prognostication of various cancers, including OS [5–7]. Elucidating the molecular mechanisms contributing to OS progression is crucial in identifying genes that have higher correlations with OS progression. Nevertheless, the comprehensive understanding of the molecular mechanism underlying OS development remains elusive.

Acyl-CoA synthetase long-chain family member 4 (ACSL4), belongs to ACSL family members, is a key Acyl-CoA synthetase enzyme that regulates lipid composition. ACSL4 plays a significant role in cell function and embryonic development [8]. Dysregulated expression of ACSL4 has been reported in various types of cancers, including colorectal cancer, ovarian cancer, and breast cancer [9–11]. It has been reported that ACSL4 is crucial for the development and progression of cancers, with its up-regulation clearly associated with tumor growth, recurrence, metastasis, and therapy resistance [12]. Furthermore, a previous study suggested that the proto-oncogenic transcriptional co-activator YAP might promote ferroptosis by regulating ACSL4, in turn involving in the cancer progression [13]. However, to our knowledge, the role and function of ACSL4 in OS remain unclear.

Herein, the expression of ACSL4 was briefly evaluated in OS samples and para-carcinoma tissues by immunohistochemical (IHC) staining. Furthermore, we employed shRNA targeting ACSL4 to silence ACSL4 to characterize the functional importance of ACSL4 in osteosarcoma cells. Afterward, the biological effects of ACSL4 knockdown on the cell proliferation, apoptosis, cell cycle, and migratory potential in vitro, as well as tumor growth in vivo were investigated using both in vitro and in vivo models. Besides, we conducted a preliminary exploration of ACSL4 downstream regulation. In summary, our findings highlighted the importance of ACSL4 in the development and progression of OS, establishing it as a promising target for future OS treatment.

## Materials and methods

### Cell lines

OS cell lines (MNNG/HOS, U-2OS, MG-63, and 143B) were purchased from ATCC and SV40T-immortalized human osteoblasts (HNOB) were purchased from the Crisprbio (Beijing, China). U-2OS cell line were cultured in MEM with 10% FBS, while MNNG/HOS and MG-63 cells were grown in media containing 89% MEM, 10% fetal bovine serum (Invitrogen), and 1% penicillin and streptomycin. 143B cells were cultured in EMEM medium supplemented with 10% FBS, 1% MEM NEAA (non-essential amino acids), and 1% penicillin/streptomycin. All cell lines were maintained in incubator containing 5% CO_2_ at 37 °C. After 48 h of transfection, cells were treated with TGF-β inhibitor at 100 uM/well.

### Immunohistochemical staining (IHC)

The tissue microarray containing 61 OS tumor tissues and 8 para-carcinoma tissues was purchased from Zhongke Guanghua (Xi’an) Intelligent Biotechnology Co., Ltd. (L714901). Informed consent was taken from all the participants and all experiments were approved by Ethical Committee of The Second Affiliated Hospital of Guangxi Medical University. All tissue samples and pathological characteristics of OS patients including age, grade, and tumor size, T Infiltrate lymphatic metastasis (N), and stage were collected with informed consent. Briefly, the paraffin-embedded tissue samples were dewaxed by xylene, dehydrated, and rinsed with ethanol. After that, Tissue sections underwent antigen repair with citrate buffer solution, followed by blocking with 3% H_2_O_2_ and then by 5% serum. Subsequently, tissue sections were incubated in the primary antibodies anti- ACSL4 (1: 100, Sanying, No. 22401-1-AP) or anti-Ki-67 (1:100, Abcam, Cat No. ab16667) solution at 4 °C overnight, washed by 1 × PBST, and then incubated with the secondary antibody solution (goat anti-rabbit IgG H&L (HRP):1:400, Abcam, No. ab97080) at 37 °C for 1 h. Subsequently, tissue sections were re-stained with hematoxylin (Baso) for 10–15 s after incubating in DAB solution in the dark for 5 min. Finally, tissue sections were separated by alcohol, sealed with neutral gum, and then photographed under a microscope (Olympus). IHC results were scored in terms of positive cells and staining intensity. IHC score = positive cell score × staining color intensity score. The higher the score, the higher the expression of protein to be detected.

### Lentivirus vector construction and cell transfection

In brief, three RNA interference (RNAi) target sequences were designed using ACSL4 gene as templates (shACSL4#1: 5′-GAGTATGTATCTCTTGGGAAA-3′; shACSL4#2: 5′-AAGCTGAAATACTGAAAGAAA-3′; and shACSL4#3: 5′-TCCGGAAATCATGGATAGAAT-3′). RNAi sequences were synthesized and then annealed to generate double-stranded DNA. Subsequently, double-stranded DNA was attached to the linearized vector by T4 DNA ligase, and the recombinant vector was then transformed into the competent Escherichia coli cells and inoculated in LB solid medium containing ampicillin. Then, the plasmids were extracted and verified by PCR and sequencing. The plasmids carrying the RNAi sequence were diluted with Opti-MEM medium, and added in Viafect transfection reagent, followed by co-infecting into 293 T cells. The shACSL4 lentivirus plasmids were extracted 72 h later. Then, shACSL4 lentiviral recombinants (carrying green fluorescent protein) were transfected into healthy growing OS cells and then cultured in an incubator at 37 °C for 18 h. 72 h after transfection, stable cells expressing lentiviral shRNA were selected with puromycin. Finally, the florescence was observed under a fluorescence microscope to evaluate the transfection efficiency.

### Real-time qPCR (qPCR)

Total RNA was abstracted from cells with Trizol (Sigma) according to the kit instruction. The cDNA fragments were synthesized by reverse transcription of RNA based on the manufacturer instruction of Hiscriprt QRT supermix for qPCR (+ gDNA WIPER) (Vazyme). Quantitative real-time PCR (qRT-PCR) were carried out with cDNA, AceQ qPCR SYBR Green master mix (Vazyme), forward and reverse primers (ACSL4 forward primer: 5′–ATTCCTCCAAGTAGACCAACGC–3′ and reverse primer: 5′–CCCAGTCCAGGTATTCTTTCACA–3′; GAPDH forward primer: 5′–TGACTTCAACAGCGACACCCA–3′ and reverse primer: 5′–CACCCTGTTGCTGTAGCCAAA–3′), and other reagents following a two-step method on the Vii7 Real-Time PCR instrument (ABI). GAPDH was used as an internal control, and the relative mRNA level of target gene is calculated by the formula 2^−△△Ct^.

### Western blotting (WB)

Cells and tissues were lysed in lysis buffer to extract total proteins, and the protein concentration was then determined using BCA Protein Assay Kit (HyClone-Pierce). After that, 20-μg equal protein was separated with 10% SDS-PAGE and then transferred to the PVDF membrane and incubated with TBST solution containing 5% skim milk at room temperature for 1 h. Subsequently, PVDF membranes were incubated with primary antibody solution overnight at 4 °C after blocked and then incubated with secondary antibody solution for 1 h at room temperature following cleaning with TBST for 3 times. Finally, visualization of PVDF membranes employed immobilon Western chemiluminescent HRP Substrate (Millipore), and chemiluminescence imaging was carried out using a Chemiluminescence get imaging system (GE). Antibodies used in western blotting assay are provided in Supplementary Table 1.

### CCK8 assay

After transfection, OS cells were harvested, digested with trypsin (Sangon Biotech (Shanghai) Co., Ltd) and then re-suspended into cell suspension. Subsequently, 2000 cells per well per 100 μL were inoculated into 96-well plate, with a total of five 96-well plates. Starting at 24 h after inoculation, 10 μL/well CCK-8 reagent was added before the end of each time point and then incubated for another 2–4 h, for 5 consecutive days. The optical density (OD) of each well cells were determined at 450 nm by a microplate reader, the cell proliferation curve was then plotted.

### Flow cytometry

Flow cytometry was performed to detect cell apoptosis and cell cycle.

For cell apoptosis, transfected MNNG/HOS and U-2OS cells were seeded into 6-well plate in triplicate separately, digested with trypsin, re-suspended into cell suspension, and collected in 5-mL centrifuge tube. After centrifuging at 1300 rmp for 5 min, the precipitated cells were washed with D-Hank’s solution and then with 1 × binding buffer once. Afterward, cells were centrifuged at 1500 rpm for 3 min and re-suspended with 1 × binding buffer. Subsequently, 5-µL Annexin V-APC (eBioscience, USA) was added to stain for 15 min in the dark. Finally, the cell apoptosis was detected by the flow cytometer (Millipore, USA). For cell cycle, transfected MNNG/HOS and U-2OS cells were seeded into 6-cm dish in triplicate separately, washed with pre-cooled PBS (4 °C, pH = 7.2), and then fixed with 70% ethanol for 1 h at 4 °C. After centrifuging, cells were washed with PBS, and then re-suspended with 1.5 mL of cell staining solution (40 × PI mother liquor: 100 × RNase mother liquor: 1 × PBS = 25:1:1000). Finally, cell cycle distribution was detected by the flow cytometer (Millipore, USA).

### Wound healing

Lentivirus-transfected OS cell suspensions were added to 96-well plate, 5 × 10^4^ cells per well. The media was changed to low concentration serum media in the next day, and the 96-well plates were gently pushed upward from the lower central part with 96 wounding replicators (VP scientific). After that, the cells were rinsed with serum-free medium3 times and then cultured in medium containing 0.5% FBS at 37 °C in an incubator containing 5% CO_2_. The plates were scanned with Cellomics (Thermo) at 0 h and 24 h. Finally, the cell migration was evaluated by analyzing cell area from the same field of view at different time points. For quantification of migration ratio, images were captured and measured the width of wound at the same site on 0 h and 24 h.

### Transwell assay

The upper chamber of Transwell chambers (corning) was incubated with 100-μL serum-free medium for 2 h. During this time, transfected MNNG/HOS and U-2OS cell lines in logarithmic growth phase were digested, re-suspended in serum-free medium, and then counted. After 24 h, the medium in upper chamber was removed, and 600 μL medium with 30% FBS was added into the lower chamber. Afterward, cell suspension was diluted with serum-free medium and then added into the upper chamber (10^5^ cells/well). Subsequently, the upper chamber was carefully transferred to the lower chamber for incubation for 24 h. In the following day, the non-metastatic cells were gently swabbed with a cotton swab and the upper chamber was then soaked in 400-μL Gimsa solution for 5 min, followed by rinsing with water and dried in air. Finally, the cell metastasis was observed and photographed using a microscope (Olympus).

### Subcutaneous tumorigenesis model in nude mice

6-week-old BALB/c nude mice (male) were purchased from Cavens Biogle Model Animal Research Co., Ltd. (license number SCXK license number SCXK 2012-0001, Suzhou, China) and kept in captivity under the following conditions: temperature, 22‑25˚C; humidity, 50–60%; and 12-h light/dark cycle. All animal experiments were approved by the medical ethics committee of Ethical Committee of The Second Affiliated Hospital of Guangxi Medical University. Nude mice were randomly divided into control group (shRNA group) and experimental group (shACSL4 group) with 5 nude mice in each group. 200-μL cell suspension (1 × 10^7^ MNNG/HOS cells infected with shACSL4 or shCtrl) was injected subcutaneously into nude mice. From day 7 onward, the long and short diameter of the tumors were measured every 4 days with a Vernier caliper, and the tumor volume was calculated to plot the tumor growth curve. Tumor volume: *V* = *π*/6 × *L* × *W*^2^. V represents tumor volume; *L* represents the long diameter for the tumor; and *W* represents the short diameter of the tumor. On the last day of the experiment, the tumors were removed from the sacrificed nude mice, weighed and measured in volume, and then photographed with a digital camera. Finally, tumor tissues were stored at − 80 °C for IHC staining and Western blot analysis.

### Immunoprecipitation (IP) assay

The cells were lysed in a pre-chilled IP lysis buffer and the protein concentration was assessed using the BCA method. Following this, the pre-cleared lysates were subjected to incubation with the primary antibody specific to the protein of interest. Subsequently, protein A/G agarose beads were added to facilitate the formation of antibody-protein complexes. After extensive washing to remove non-specifically bound proteins, the protein complexes were eluted with 6 × loading buffer and then analyzed using western blotting.

### Statistical analysis

Sign test were employed to evaluate whether ACSL4 gene expression was statistically different between OS tissues and para-carcinoma tissues. Chi-square Test and Mann–Whitney U analysis were used to analyze the signification of ACSL4 expression at different levels in different pathological characteristics, and Spearman correlation analysis was conducted to analyze the correlation between the expression level of ACSL4 in cancer tissues and pathological characteristics. Statistical analysis was performed using one-way ANOVA, followed by Student t test. *P* < 0.05 represented significant difference.

## Results

### Expression of ACSL4 in OS tissues and cells

**Fig. 1:**
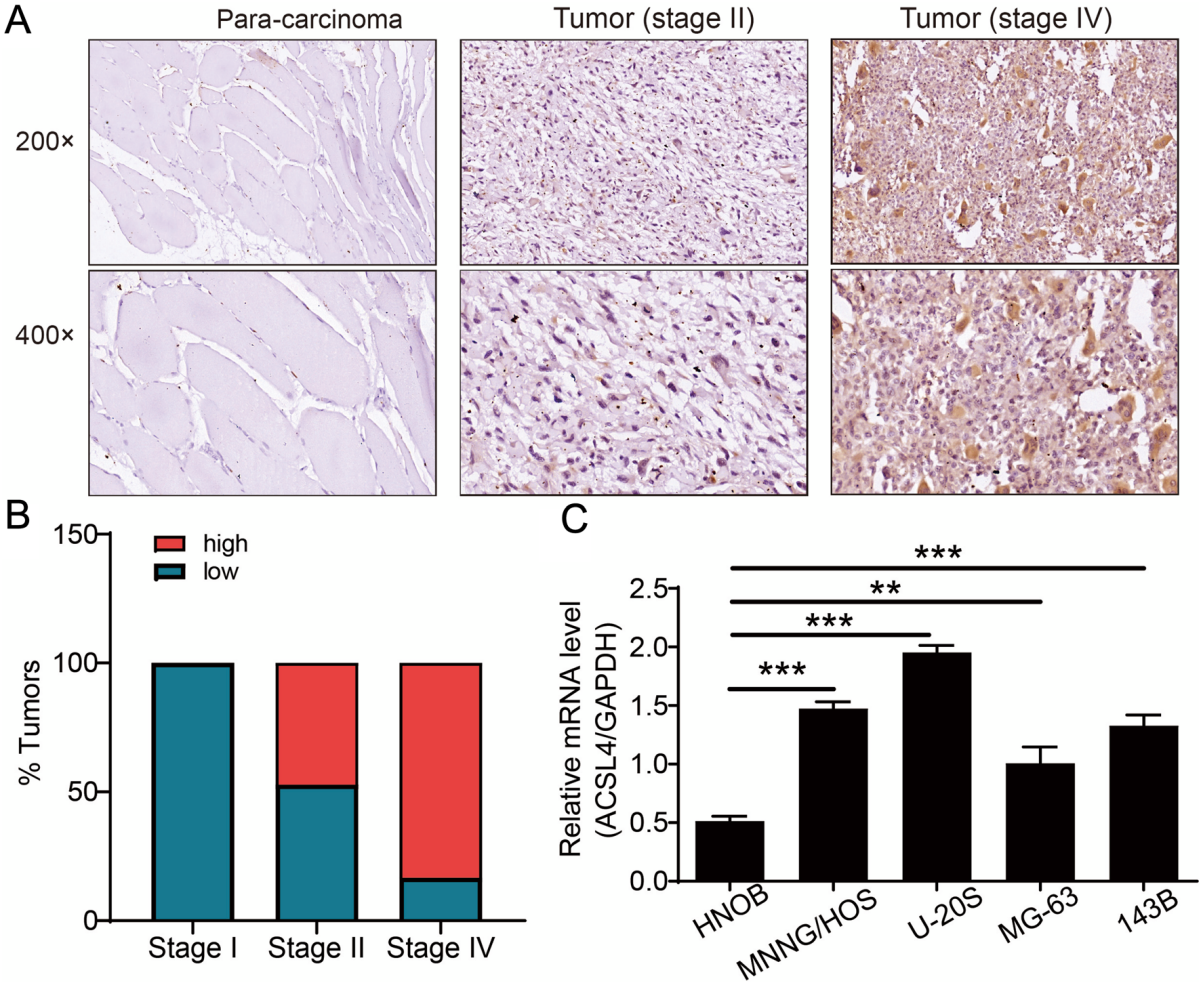
Expression of ACSL4 was elevated in OS. **A** OS tissues exhibited the strong positive expression of ACSL4, while weak positive expression in para-carcinoma tissues. Scale bar = 50 μm. **B** The percentage of high ACSL4 expression and low ACSL4 expression in stage I–IV OS. **C** The expression of ACSL4 in the OS cell lines (MNNG/HOS, U-2OS, MG-63, and 143B) and SV40T-immortalized human osteoblasts (HNOB) was detected by qPCR assay. Results were presented as mean ± SD. ***P < 0.001

To explore the roles of ACSL4 in OS, the expression pattern of ACSL4 was contrasted in OS tumor tissues and para-carcinoma tissues by IHC assays. As shown in Fig. 1, OS tumor tissues was strong positive for ACSL4 as compared to para-carcinoma tissues (Table 1). Next, we further analyze the relationship between the expression level of ACSL4 and the clinicopathological parameters in patients with OS. It can be found that the expression of ACSL4 was different among the different tumor stages of OS patients (Table 2). As suggested by Spearman rank correlation analysis, the expression level of ACSL4 had a significant positive correlation with stages (Table 3). Specifically, the percentage of stage IV OS cases with high ACSL4 expression (83.33%) was markedly greater than that of stage I OS (47.17%) (Fig. 1B). Furthermore, we investigated the levels of ACSL4 mRNA expression in OS cell lines (MNNG/HOS, U-2OS, MG-63, and 143B) and SV40T-immortalized human osteoblasts (HNOB). The results indicated that the OS cell lines displayed higher levels of ACSL4 mRNA expression compared to HNOB (Fig. 1C), implying a possible link between ACSL4 and OS progression.

**Table 1:** Expression patterns in gastric cancer tissues and para-carcinoma tissues revealed in immunohistochemistry analysis

ACSL4 expression	Tumor tissue		Para-carcinoma tissue		*P* value
	Cases	Percentage (%)	Cases	Percentage (%)	
Low	44	72.1	8	100	< 0.001
High	17	27.9	0	0

**Table 2:** Relationship between ACSL4 expression and tumor characteristics in patients with osteosarcoma

Features	No. of patients	ACSL4 expression		*P* value
		Low	High	
All patients	61	44	17	
Age (years)				0.527
< 28	30	14	16	
≥ 28	31	17	14	
Gender				0.242
Male	41	23	18	
Female	20	8	12	
Tumor size				0.975
≤ 6 cm	15	7	8	
> 6 cm	26	12	14	
T Infiltrate				0.700
T1	29	13	16	
T2	27	17	10	
T3	5	1	4	
Lymphatic metastasis (N)				0.081
N0	55	30	25	
N1	6	1	5	
Stage				0.031
1	2	2	0	
2	53	28	25	
4	6	1	5

**Table 3:** Relationship between ACSL4 expression and tumor characteristics in patients with osteosarcoma

		ACSL4
Stage	Spearman correlation	0.278
	Significance (two-tailed)	< 0.05
	N	61

### Establishment of ACSL4 knockdown cell model

**Fig. 2:**
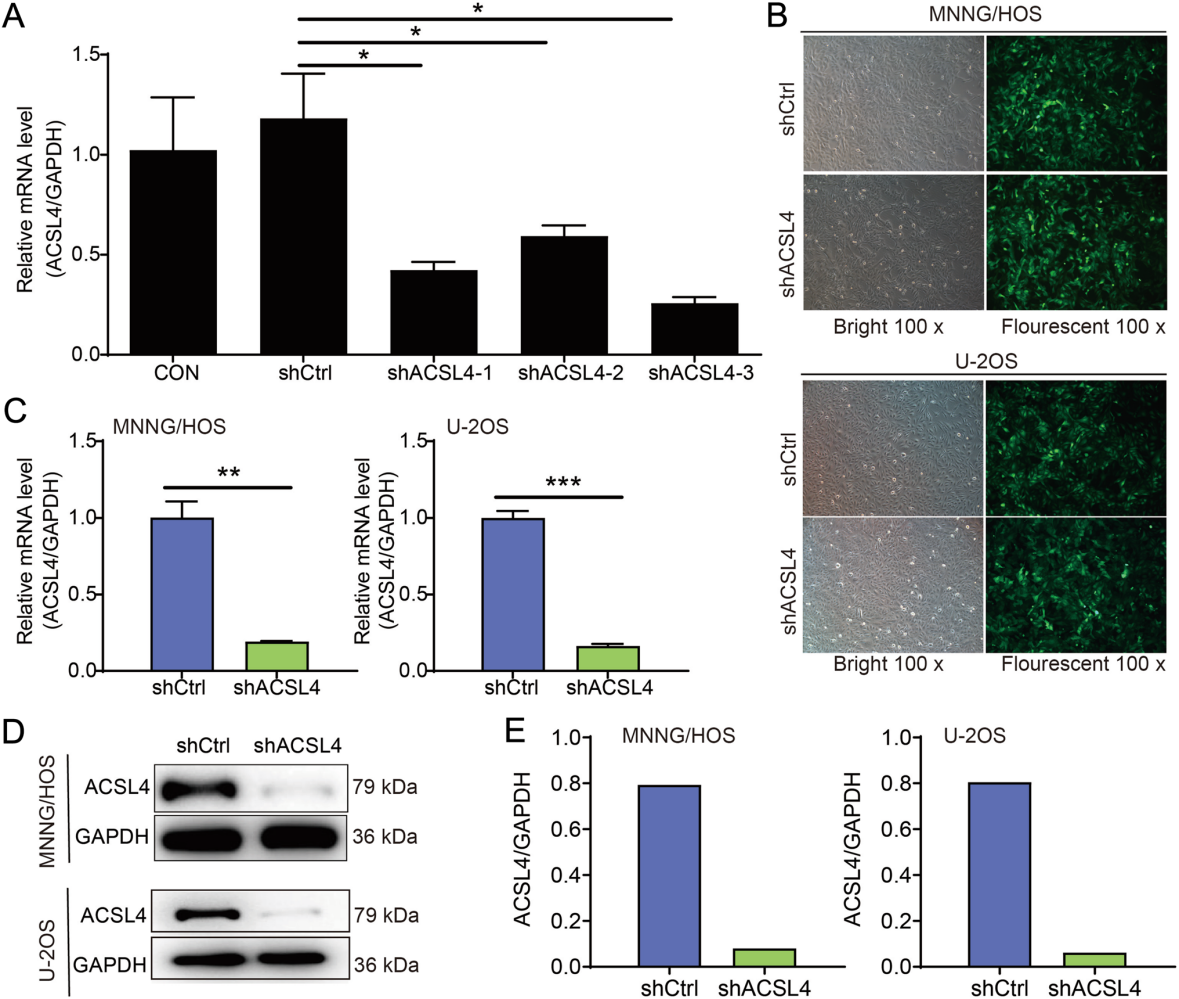
Construction of stable ACSL4 knockdown cell models in OS cell lines. **A** The best effect sequence of knockdown was screened by qPCR analysis. **B** Successfully transfected OS cells were tagged with green fluorescent protein (GFP). Magnification, ×200. **C** The knockdown efficiency of ACSL4 in MNNG/HOS and U-2OS cells transfected with shACSL4 or shCtrl was evaluated by qPCR analysis. **D** The knockdown efficiency of ACSL4 in MNNG/HOS and U-2OS cells transfected with shACSL4 or shCtrl was verified by Western blot. **E** The quantification of ACSL4 protein expression. GAPDH was used as the loading control. Results were presented as mean ± SD. *P < 0.05, **P < 0.01, and ***P < 0.001

Next, ACSL4 knockdown cell models was established in OS cells to assess the biological effects of ACSL4 on OS. Briefly, the MNNG/HOS cells were stably transfected with shACSL4-1, shACSL4-2, and shACSL4-3, while cells transfected with shCtrl were used as negative controls. As shown in Fig. 2A, shACSL4-3 (78.2%) possessed the best knockdown effect among three shRNAs, which was used in the following functional experiments. Afterward, MNNG/HOS and U-2OS cells were transfected with shCtrl or shACSL4, and successfully transfected cells were confirmed through visualization of green fluorescent protein (GFP) expression (Fig. 2B). Besides, the knockdown efficiency of ACSL4 was assessed by Western blot analysis assay and qPCR assay at both the protein and mRNA levels. As expected, knockdown efficiency of ACSL4 was 80.91% in MNNG/HOS cells and 83.70% in U-2OS cells, when compared to the shCtrl cells (Fig. 2C). Simultaneously, the results of Western blot further confirmed that ACSL4 was effectively knocked down in the both OS cell lines (Fig. 2D, E). Altogether, these results suggested the successful establishment of ACSL4 knockdown models in MNNG/HOS and U-2OS cell lines that used for the following experiments.

### *The effects of ACSL4 knockdown on malignant biological behavior of OS *in vitro

**Fig. 3:**
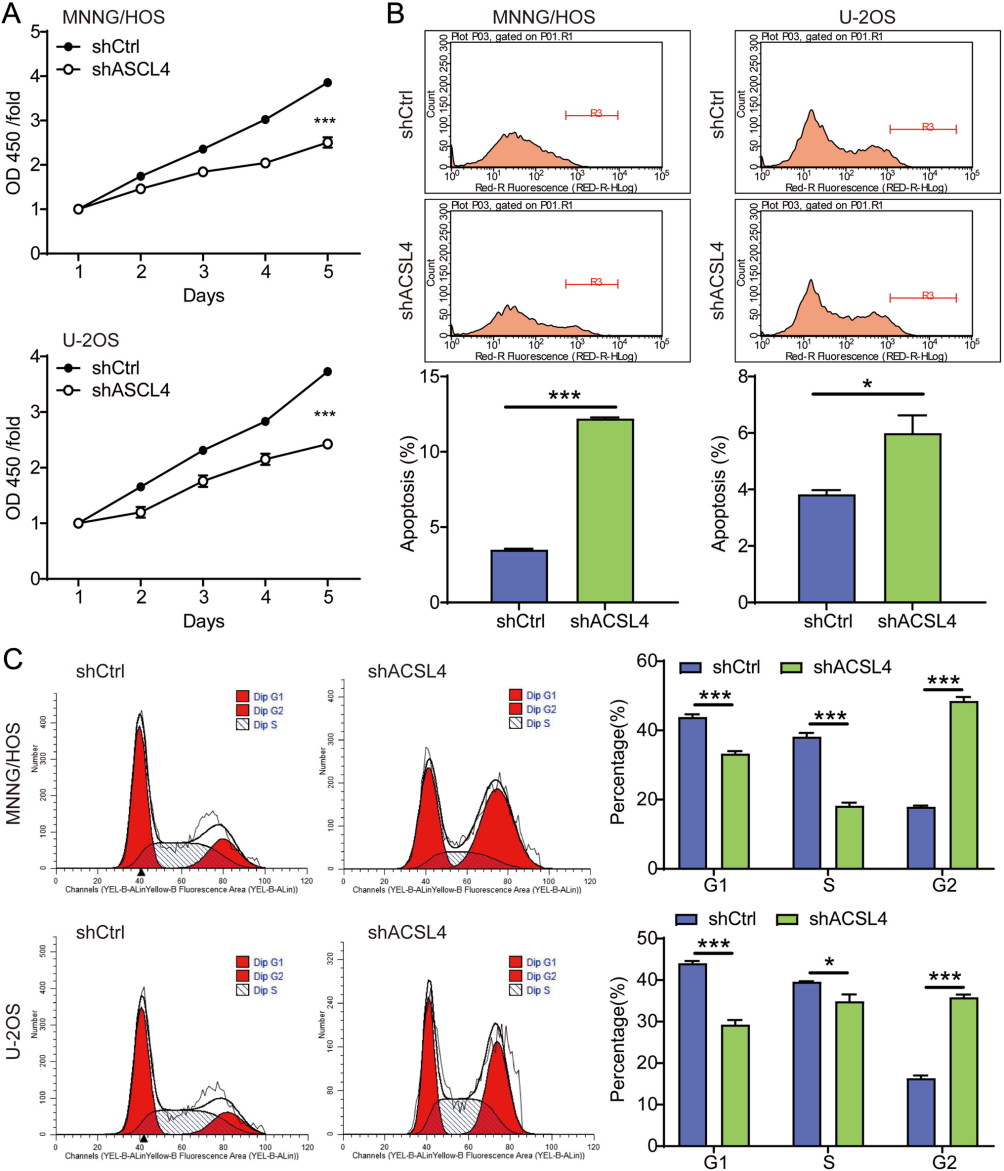
ACSL4 downregulation suppressed OS cell proliferation and induced apoptosis and cycle arrest. **A** The effect of ACSL4 knockdown on cell proliferation in MNNG/HOS and U-2OS cells was revealed by CCK-8 assays. **B** The effect of ACSL4 knockdown on cell apoptosis in MNNG/HOS and U-2OS cells was assessed by the flow cytometry. **C** The effect of ACSL4 knockdown on cell cycle distribution in MNNG/HOS and U-2OS cells was also evaluated by the flow cytometry. Results were presented as mean ± SD. *P < 0.05 and ***P < 0.001

The biological functions of ACSL4 knockdown were elucidated through cell proliferation, cell cycle, apoptosis, and cell migration in OS cells in vitro. The results from CCK8 assay showed that MNNG/HOS and U-2OS cells with ACSL4 deletion exhibited slower proliferation compared to the shCtrl group (Fig. 3A). Given that apoptosis and cell cycle arrest often contribute to decreased cell proliferation, the effects of ACSL4 knockdown on the apoptosis and cell cycle were analyzed by flow cytometry. Compared with shCtrl group, silencing ACSL4 significantly accelerated cell apoptosis in both OS cell lines (Fig. 3B). Furthermore, the percentage of OS cells in S phase was decreased, while increased markedly in G2 phase upon silencing ACSL4, proving ACSL4 deficiency induced cell cycle arrest at the G2 phase in OS cells (Fig. 3C).

**Fig. 4:**
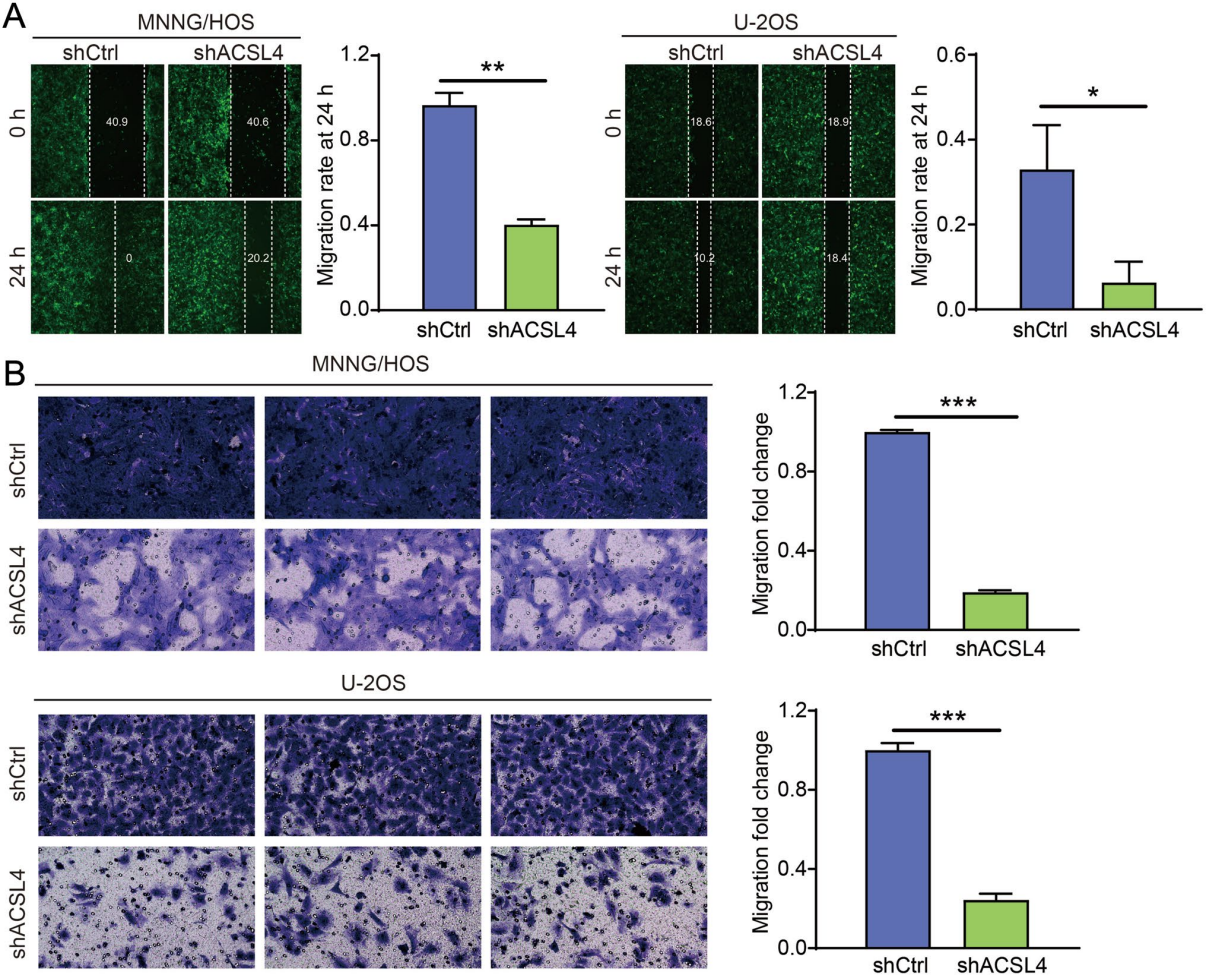
ACSL4 downregulation suppressed OS cell migration. **A** The effect of ACSL4 knockdown on cell migration rate was examined in MNNG/HOS and U-2OS cells by wound healing assay. **B** The inhibitory effect of ACSL4 knockdown on OS cell migration rate was validated by transwell assay. Results were presented as mean ± SD. *P < 0.05 and ***P < 0.001

Results from wound healing assay showed that the distance between the scratches was significantly lower in MNNG/HOS and U-2OS cells transfected with shCtrl, while ACSL4 knockdown largely lower the speed of wound healing at 24 h (Fig. 4A), indicating ACSL4 depletion suppressed the migration ability of OS cells. Additionally, similar results were found in transwell assay that ACSL4 deficiency decreased the cell migration rate of MNNG/HOS and U-2OS cell lines by 81% and 75.7%, respectively (Fig. 4B). Altogether, these above findings suggest that ACSL4 plays a crucial role in migration malignant biological behavior of OS in vitro.

### The effects of ACSL4 knockdown on OS tumor growth in vivo

**Fig. 5:**
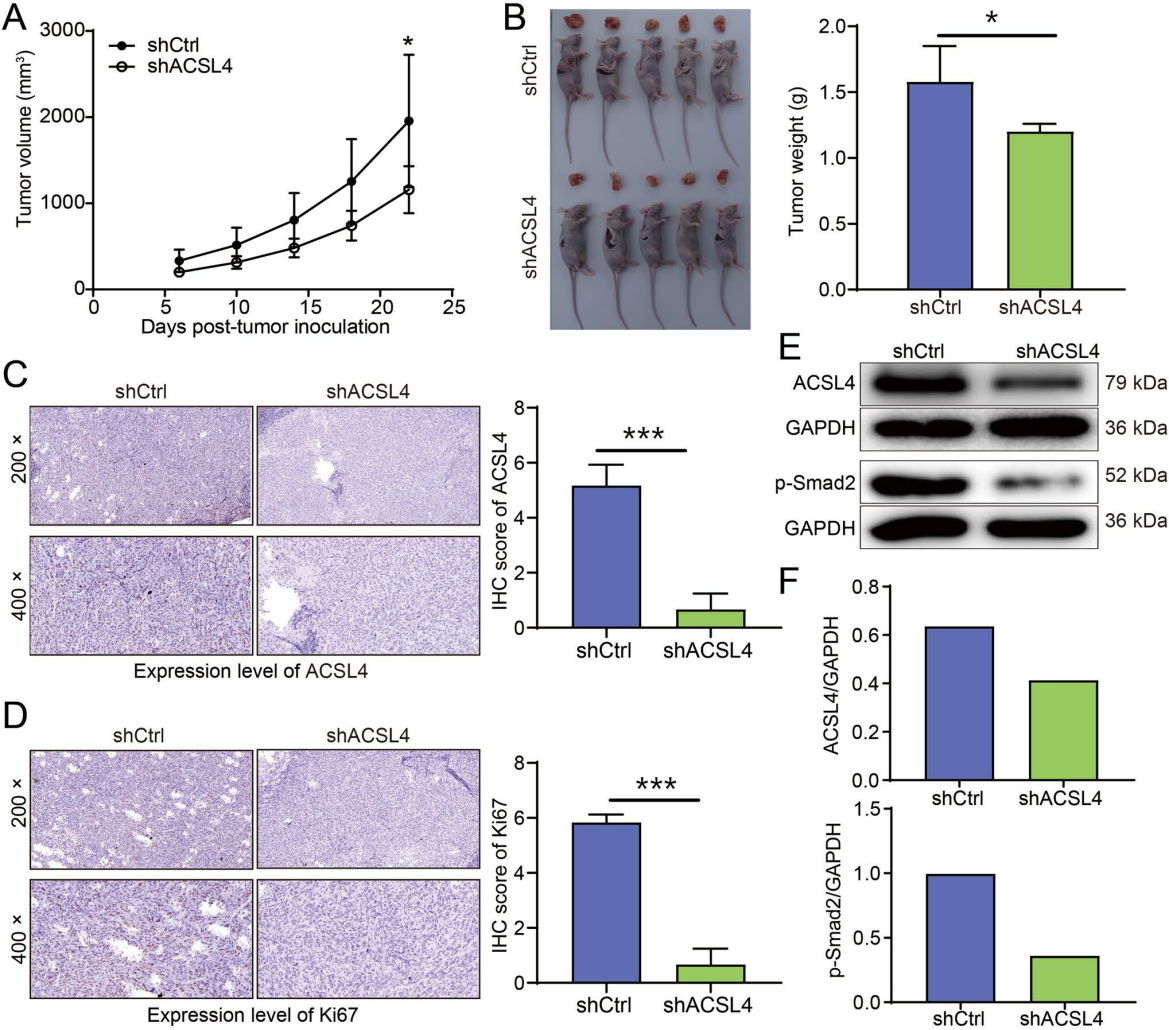
ACSL4 knockdown suppressed tumor growth in xenograft models in vivo. **A** Tumor growth curves showed that ACSL4 depletion suppresses tumor growth. **B** Representative photographs of mice and isolated tumor tissues in shCtrl and shACSL4 groups, and quantitation of tumor weight from xenograft mouse models. **C** Representative images and scoring of IHC staining for ACSL4 in tumor sections. **D** Representative images and scoring of IHC staining for Ki-67 in tumor sections. **E** Western blotting was performed to determine the relative protein expression of ACSL4 and the level of p-Smad2 in tumor tissues. **F** Quantitative analysis of protein expression, with GAPDH utilized as the loading control. Results were presented as mean ± SD. **P < 0.01

Next, subcutaneous xenograft models were constructed in nude mice to further determine the function of ACSL4 knockdown on tumorigenesis in vivo. As shown in Fig. 5A, the tumor growth in the shACSL4 group was significantly slower than that in shCtrl group. Additionally, tumors of ACSL4-depleted mice were also smaller and lighter than the tumors of shCtrl mice (Fig. 5B). Consistent with these findings, the results of IHC staining showed that the expression of ACSL4 and Ki-67 (a cell proliferation marker) was downregulation by ACSL4 knockdown (Fig. 5C, D). As expected, Western blot results showed that the protein expression of ACSL4 in the tumor tissues of shACSL4 mice was also down-regulated, compared with that of shCtrl mice (Fig. 5E). Taken together, these above results strongly supported the suppressive role of down-regulated ACSL4 against tumor development and progression in vivo. Of note, previous studies have highlighted the pivotal role of Smad2 in OS progression [14, 15]. Interestingly, the tissue from OS mice in the shACSL4 group displayed a decrease in p-Smad2 level, suggesting a potential association between ACSL4 and Smad2 signaling pathways in OS.

### Inhibition of TGF-β impaired the promoting effects of ACSL4 on the malignant phenotypes of OS cells

**Fig. 6:**
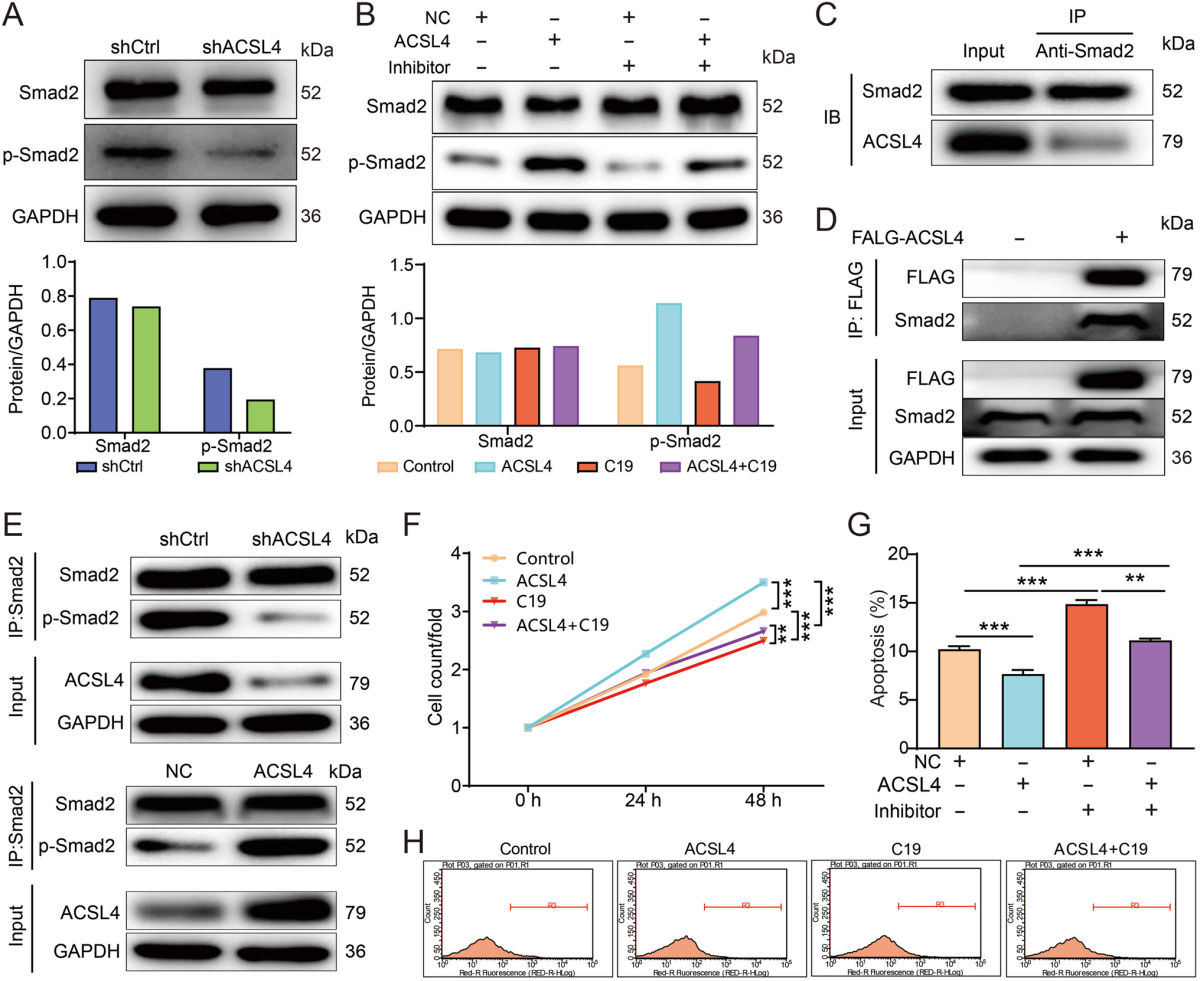
ACSL4 facilitated OS progression via targeting TGF‑β signaling pathway. **A** The protein and phosphorylation levels of Smad2 were detected and quantified by Western blot. **B** The protein and phosphorylation of Smad2 were detected in U-2OS cells with or without TGF‑β inhibitor treatment. **C** The binding between ACSL4 and Smad2 was explored in U-2OS cells. Proteins extracted from U-2OS were immunoprecipitated with anti-Smad2, and the resulting complexes were detected using anti-ACSL4. **D** Co-transfection of FALG-ACSL4 and mock plasmids into U-2OS cells allowed for the immunoprecipitation of FLAG-tagged protein and Smad2 using anti-FLAG, followed by Western blot analysis. **E** The U-2OS cells were subjected to immunoprecipitation with anti-Smad2, following stable knockdown of ACSL4 or ACSL4 overexpression, and the resulting immunoprecipitated complex was identified using anti-p-Smad2 in western blot analysis. **F** CCK8 assay was employed to show the effects of ACSL4 on cell proliferation in U-2OS cells with or without TGF‑β inhibitor treatment. **G** Flow cytometry was carried out to assess the effect of ACSL4 knockdown on cell apoptosis in U-2OS cells with or without TGF‑β inhibitor treatment. **H** Representative images of cell apoptosis by flow cytometry analysis. Results were presented as mean ± SD. **P < 0.01 and ***P < 0.001

To further investigate the regulatory effect of ACSL4 on Smad2, we knocked down or overexpressed ACSL4 in cells and monitored the expression levels of Smad2 and p-Smad2. It was found that knocking down ACSL4 could suppress the level of p-Smad2, while overexpression promoted the level of p-Smad2 (Fig. 6A, B). The binding between ACSL4 and Smad2 was further confirmed in U-2OS cells (Fig. 6C, D). Additionally, the immunoprecipitation assay provided strong evidence that overexpressing ACSL4 in U-2OS cells significantly augmented the level of p-Smad2, while ACSL4 knockdown resulted in a noticeable decrease in the phosphorylation of Smad2 (Fig. 6E). Overall, these findings indicated that ACSL4 plays a role in regulating the phosphorylation of Smad2, potentially through its interaction with Smad2.

More importantly, as previously mentioned, Smad2 is a critical factor in the TGF-β signaling pathway and can be phosphorylated by TGF-β [16]. In our study, we observed that treatment of C19 (EMT inhibitor-1), a TGF‑β inhibitor [17], decreased the level of phosphorylated Smad2 and weakened the elevation of phosphorylated Smad2 induced by ACSL4 overexpression (Fig. 6B). Based on these findings, we hypothesized that ACSL4 regulated the progression of OS by modulating the TGF‑β/Smad2 signaling pathway, which prompted us to perform a preliminary validation study using cell lines. As shown in Fig. 6F–H compared to the shCtrl group cells, ACSL4 overexpression increased the proliferation capacity of OS cells while suppressing cell apoptosis. Conversely, treatment with C19 impaired cell proliferation and increased the proportion of apoptotic cells in both cell lines (Fig. 6F, G). Interestingly, C19 strongly diminished the promoting effect of ACSL4 on cell proliferation and enhanced the suppression of ACSL4 on cell apoptosis (Fig. 6F, G). These results provided compelling evidence that ACSL4 regulates the progression of OS by targeting the TGF-β/Smad2 signaling pathway.

## Discussion

Recently, targeted therapies have improved outlook for OS patients somewhat [18–20]. Whereas, novel targets of OS for treatment up to date are still required to be identified. In this study, it was first determined the high expression of ACSL4 in OS tissues compared to para-carcinoma tissues and shed light on the positively correlation between the expression level of ACSL4 and tumor stages of OS patients. Furthermore, ACSL4 knockdown suppressed OS cell malignant phenotypes in vitro and tumor growth in vivo. Importantly, the oncogenic effects of ACSL4 overexpression on OS cells were reversed by treatment with C19, a TGF-β inhibitor. To summarize, these findings suggested that ACSL4 might be a promising target for OS therapy.

Extensive studies have shown that ACSL4 is upregulated in many types of human cancers and is involved in many biological functions, including cancer cell proliferation [21–23]. In the present study, IHC staining of OS tissues proved that the presence of elevated ACSL4 in OS tissues, with its expression level increasing in accordance with tumor malignancy. Consequently, loss-of-function experiments were performed to investigate the functional role of ACSL4 in OS progression. We observed that ACSL4 knockdown impaired the abilities of cell proliferation and induced cells arrested in G2 phase in OS cell lines. Additionally, a previous research by Killion et al. has suggested that adipocyte expression of ACSL4 was associated with increased activation of apoptosis-related factor p53 [24]. Therefore, we next detected the ability of ACSL4 to stimulate apoptosis and found that inhibition of ACSL4 accelerated the apoptosis rate of OS cells. Furthermore, there is compelling evidence supporting a positive association between ACS L4 expression level and the migration and invasion capabilities of breast cancer cells [25]. Likewise, our results indicated that ACSL4-deficient OS cells displayed reduced migration velocity. It is also encouraging that ACSL4 deletion effectively reduced the rate of tumor growth in xenograft tumor model. To further validate these findings, we performed in vitro experiments to examine the effects of ACSL4 overexpression. As anticipated, ACSL4 overexpression promoted OS cell proliferation while reducing cell apoptosis. These findings suggested that ACSL4 might play an important role in the progression of OS. However, the underlying molecular mechanisms through which ACSL4 regulated OS needed further exploration.

The TGF-β/Smad signaling pathway has been shown to participate in osteogenic differentiation [26] and is closely associated with OS development and progression [27, 28]. Smad2 was identified as a primary mediators of TGF-β signaling, which might be phosphorylated upon TGF-β stimulation [29, 30]. Our study revealed that ACSL4 might positively regulate the phosphorylation of Smad2 through its interaction with Smad2, implying that up-regulation of ACSL4 likely resulted in the activation of Smad2 in OS. Moreover, we observed that TGF-β inhibitor C19 suppressed ACSL4-mediated phosphorylation of Smad2. As the TGF-β signaling pathway is known to regulate various cellular processes in cancer, including cell growth, cell differentiation, and apoptosis [31], we thus hypothesized that ACSL4 contributed to OS cell progression by regulating TGF‑β/Smad2 signaling pathway. To validate our hypothesis, we conducted functional experiments and demonstrated that the application of C19, a TGF-β inhibitor, significantly reduced the proliferation capacity and promoted apoptosis in OS cells. More importantly, C19 attenuated the promoting effect of ACSL4 overexpression on cell proliferation and restored the level of apoptosis in OS cells. The above results strongly suggested that ACSL4 regulated OS cell proliferation and apoptosis via targeting TGF-β/Smad2 signaling pathway.

While this study provides important insights into the role and function of ACSL4 in OS, there are specific areas that require further investigation and understanding. Firstly, it should be noted that TGFβ signaling could induce epithelial–mesenchymal transition (EMT) through Smad activation [32]. Nevertheless, the exact role and mechanism by which ACSL4 regulates EMT in OS through targeting of the TGF-β/Smad2 signaling pathway requires further investigation. Additionally, due to the complexity of the interactions and cross-talk involved in TGF-β/Smad2 activation, a more comprehensive study is needed to elucidate the specific mechanisms by which ACSL4 modulates the TGF-β/Smad2 signaling pathway, thus unveiling its role in the development of OS. Furthermore, while ACSL4 has been discovered to influence the lipid composition required for execution of ferroptosis, which is considered as a determinant of ferroptosis sensitivity [33, 34], the effect of ACSL4 on ferroptosis in OS remains to be fully characterized.

In conclusion, our study revealed up-regulation of ACSL4 expression in OS. Depletion of ACSL4 significantly inhibit the malignant phenotype of OS cells, while its overexpression exerted the opposite effect. Additionally, ACSL4 was found to exert a significant influence on the phosphorylation of Smad2, and the effects of ACSL4 up-regulation on OS cell proliferation and apoptosis were blocked by a TGF-β inhibitor. These findings strongly support the notion that ACSL4 may serve as a new biomarker of OS that influences the progression of the disease via modulating TGF-β/ Smad2 signaling pathway.

## Supplementary Information

Below is the link to the electronic supplementary material.

Supplementary file1 (DOCX 13 KB)

## Data Availability

The dataset supporting the results of this article are included within the article.
