# Supplementary material for: ACSL4 accelerates osteosarcoma progression via modulating TGF-β/Smad2 signaling pathway
Source: Mol Cell Biochem. 2024 Apr 2;480(1):549–62. doi: 10.1007/s11010-024-04975-5 (PMC11695466; doi:10.1007/s11010-024-04975-5)
Supplement: Supplementary file 1 — Supplementary file1 (DOCX 13 KB) [file 11010_2024_4975_MOESM1_ESM.docx]

**Table S1** Antibodies applied in Western blot of this study.

| Primary antibody | Size/kDa | Diluted multiples | Source | Company | Catalog No. |
| --- | --- | --- | --- | --- | --- |
| ACSL4 | 79 | 1:2000 | Rabbit | Abcam | ab155282 |
| Smad2 | 52 | 1:1000 | Rabbit | Proteintech | 12570-1-AP |
| p-Smad2 | 52 | 1:1000 | Rabbit | Abcam | ab280888 |
| GAPDH | 36 | 1:30000 | Mouse | Proteintech | 60004-1-lg |
| Secondary antibody |  | Diluted multiples |  | Company | Catalog No. |
| Goat Anti-Rabbit |  | 1:3000 |  | Beyotime | A0208 |
| Goat Anti-Mouse |  | 1:3000 |  | Beyotime | A0216 |
